# Prediction of birthweight with early and mid-pregnancy antenatal markers utilising machine learning and explainable artificial intelligence

**DOI:** 10.1038/s41598-025-11837-7

**Published:** 2025-07-19

**Authors:** Manohar Pavanya, Krishnaraj Chadaga, Vennila J, Akhila Vasudeva, Bhamini Krishna Rao, Srikanth Prabhu, Shashikala K Bhat

**Affiliations:** 1 Department of Obstetrics and Gynecology,Dr TMA Pai Hospital (Udupi), Melaka Manipal Medical College, Manipal Academy of Higher Education, Manipal, Karnataka India; 2 Manipal Institute of Technology, Manipal Academy of Higher Education, Manipal, Karnataka India; 3 Statistics, Manipal College of Health Professions, Manipal Academy of Higher Education, Manipal, Karnataka India; 4 Division of Fetal Medicine Department of Obstetrics and Gynaecology, Kasturba Medical College, Manipal Academy of Higher Education, Manipal, Karnataka India; 5 Department of Physiotherapy, Manipal College of Health Professions, Manipal Academy of Higher Education, Manipal, Karnataka India

**Keywords:** Low birthweight, Explainable artificial intelligence, Antenatal care, Machine learning, Medical research, Experimental models of disease, Outcomes research, Health care, Diagnosis, Risk factors, Engineering, Biomedical engineering

## Abstract

Low birthweight (LBW) is a significant health challenge worldwide, as these neonates experience both short- and long-term disabilities. Factors affecting maternal and fetal health during early to mid-pregnancy can greatly influence fetal development. Prediction of birthweight using machine learning (ML) models with antenatal data may help in better clinical management. However, the lack of explainability in these models has raised concerns within the medical community. To address this issue, our study aims to develop a more practical ML model by incorporating explainable artificial intelligence (XAI). We prospectively collected real-world clinical data of 19 maternal and fetal clinical features from 237 singleton pregnancies. Statistical analyses were conducted using Jamovi (version: 2.6.26) and JASP team (2024) JASP (version: 0.18.3). Multiple ML classifiers were employed. We developed a stacked ensemble model that integrated various algorithms, including a custom-stacked ensemble approach and three XAI methodologies: Shapley Additive Explanations (SHAP), Local Interpretable Model-agnostic Explanations (LIME), and Anchor. These methods provided meaningful explanations to help construct reliable and optimal clinical predictive models. Among the ML classifiers evaluated, the AdaBoost model achieved the highest performance, with a maximum accuracy of 77%, a precision of 73%, a recall of 77%, and an F1 score of 72%. The stacked model demonstrated an accuracy of 75%, indicating its possibility in clinical application. However, the accuracy of these models might be affected by the limited dataset, which included pregnant women undergoing treatment for thyroid abnormalities, diabetes, and hypertension. Our developed model identified several key attributes that influence birthweight, such as maternal height, nuchal translucency thickness, parity, crown-rump length, glycated hemoglobin, hypertensive disorders of pregnancy, and pregnancy-associated plasma protein A. This model can assist medical professionals in making more precise birthweight predictions using routinely collected antenatal parameters, enabling timely medical decisions and treatments.

## Introduction

Birthweight is a key index of maternal–fetal wellness and reflects the health profile of the newborn. Several factors influence fetal growth, including maternal dietary intake, circulatory system function, uteroplacental function, thyroid function, genetic conditions, and various risk factors such as diabetes, hypertension, and medical history^1^. Sustainable Development Goal 3.2 (SDG) aims to eliminate preventable neonatal deaths and requires all countries to reduce their neonatal mortality rate by 2030^2^. Globally, about one in seven neonates is born as a low-birthweight baby (LBW)^3,4^.

**Fig. 1:**
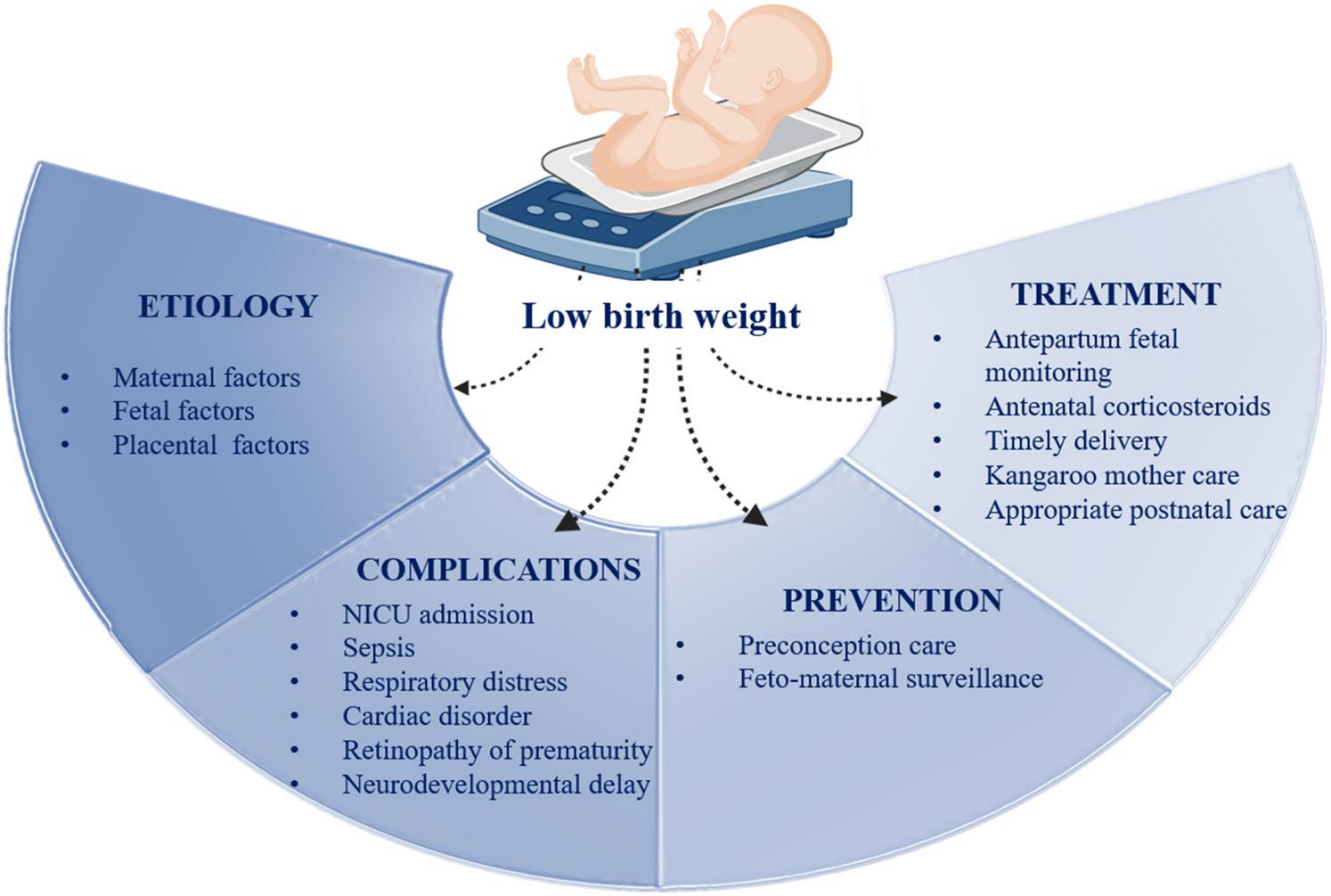
Illustration of etiology, complications, prevention, treatment, and management of LBW.

The World Health Organization (WHO) classifies a birthweight of less than 2500 g as LBW, presenting a significant global challenge with both short and long-term complications^4,5^. LBW is further categorized into very low birthweight (VLBW), weighing less than 1500 g, and extremely low birthweight, which is less than 1000 g^6^. An estimated 19.8 million live newborns are classified as LBW, with majority of this population residing in Southern Asia and Sub-Saharan Africa^7^. Data from the National Family Health Survey indicates that the prevalence of LBW in India decreased from 22% in 2005–2006 to 17.5% in 2015–2016^8,9^. Figure 1 illustrates the etiology, complications, prevention, treatment, and management of LBW.

LBW can result from various factors, including preterm birth, being small for gestational age (SGA), and intrauterine growth restriction (IUGR), or a combination of these issues. The causes of LBW are multifactorial and may include maternal age, low maternal weight and height, anemia, birth order, smoking history, socioeconomic status, short birth intervals, multiple pregnancies, poor nutrition, and inadequate preconception care, especially in developing and low-income countries. LBW newborns are at higher risk of short-term complications like respiratory distress, hypothermia, sepsis, feeding difficulties, intraventricular haemorrhage and necrotising enterocolitis. Even if VLBW neonates survive, they face an increased risk of long-term complications, such as neurodevelopmental delays, cognitive deficits, and disorders affecting the respiratory, cardiovascular, and immune systems^10–14^. VLBW babies have more risk of perinatal morbidity and mortality as compared to LBW neonates too. A meta-analysis has shown that neonates weighing less than 2.5 kg have a 45% higher risk of developing type 2 diabetes compared to those weighing more than 2.5 kg^15^. Currently, fetal biometry measurements acquired during ultrasound aid in the estimation of fetal weight. Identifying fetuses at risk of LBW helps in treatment planning and early intervention, both of which can improve postnatal outcomes^16^.

Researchers are exploring multidisciplinary approaches that integrate artificial intelligence (AI) tools with medical data. These predictive models aid in improving patient care and diagnostic accuracy^17^. Table 1 briefly describes the previous studies on birthweight prediction. Supervised and unsupervised machine learning (ML) algorithms are being deployed to develop these models. These algorithms learn from a training dataset, identify relevant features, and make predictions based on the patterns in the new data^18^. A key consideration when implementing ML algorithms in clinical data is evaluating their effectiveness compared with the insights and judgments provided by medical professionals^19^.

**Table 1:** Description of previous studies on LBW prediction.

Reference	Objective	Algorithms used	Outcome
Reza, T.B. et al.^20^	LBW feature selection and prediction using ML	Boruta algorithm and Wrapper method Several ML classifiers	The wrapper method is the most effective way to select features. RF classification worked best for classification
Ranjbar A et al.^21^	LBW prediction with ML	8 different learning models	Classification with XGBoost outperformed all others
de Morais FL.et al.^22^	LBW prediction with tree-based ML model	5 ML classifiers	Attribute selection and eliminating duplicate data improves the model
Naimi, A.I et al.^23^	Fetal growth prediction with ML	Regression-based and data mining techniques	Smoking while pregnant raised the chances of SGA
Tao, J., et al.^24^	Fetal birthweight prediction by a temporal ML method	Convolutional Neuron Networks (CNN), RF, Linear-Regression, Support Vector Regression (SVR), Back Propagation Neural Network (BPNN), and hybrid-LSTM	Hybrid-LSTM has the highest accuracy of 93.3
Rubaiya et al.^25^	Unravelling birthweight determinants: Integrating ML, spatial analysis, and district-level mapping	Regression tree	Creates maps at the district level for regions that are at high risk of LBW

**Fig. 2:**
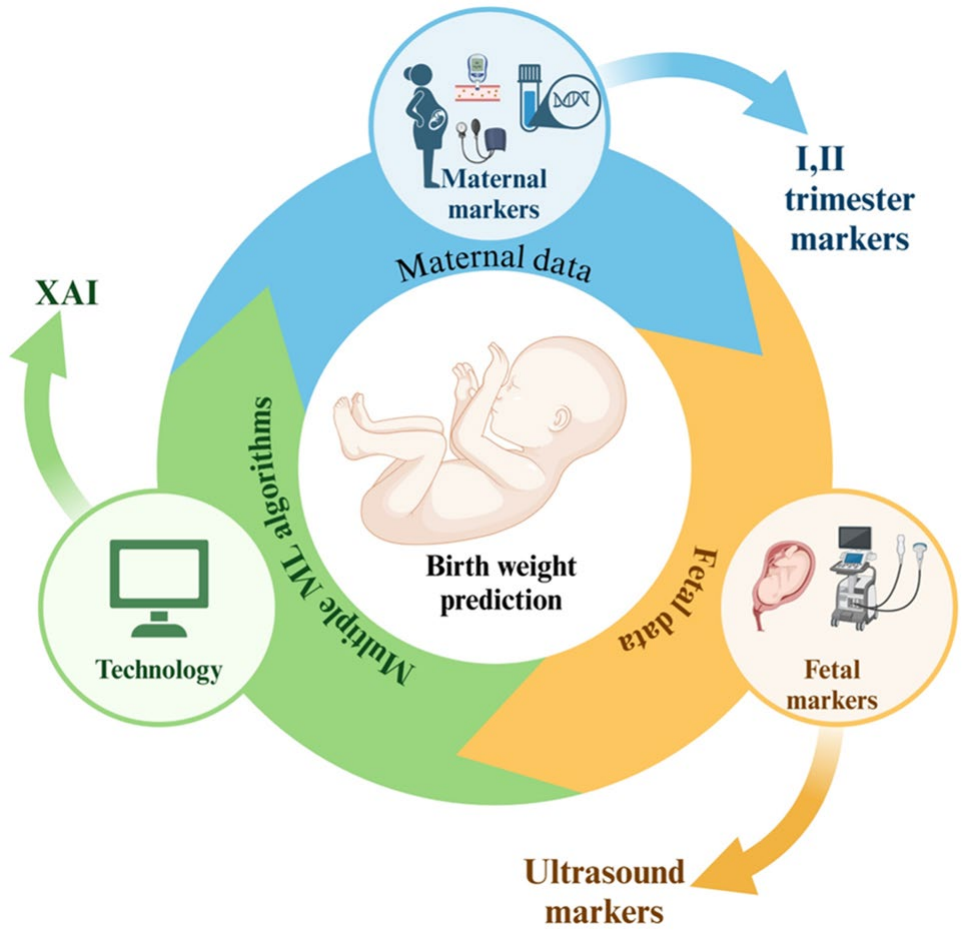
Shows the interdisciplinary approach used to predict birthweight.

To address the limitations of traditional AI models, explainable artificial intelligence (XAI) is gaining traction in predictive modelling. XAI aims to enhance model transparency, effectiveness and makes them accessible to nonexperts in various settings. The popularity of XAI has been increasing in response to the AI “black box” dilemma, which seeks to improve transparency. XAI methods facilitate communication between humans and AI systems. Furthermore, AI-based healthcare systems hold great promise for meeting the increasing demand for high-quality medical services^26^. Figure 2 illustrates our interdisciplinary framework for predicting birthweight.

In our study, a total of 19 clinically important features, as detailed in Table 2, were systematically selected and collected prospectively during early and mid-pregnancy from 237 pregnant women to develop a model for predicting birthweight. A stacked ensemble model, along with XAI techniques, was developed to classify birthweight into three categories: normal birthweight (NBW), low birthweight (LBW), and very low birthweight (VLBW). This three-class classification was chosen because the risk of neonatal complications increases as birthweight decreases^27^. Therefore, a multiclass classification approach was considered clinically appropriate for the analysis.

**Table 2:** Description of maternal and fetal features with target variable chosen in the study.

S. no.	Antenatal markers	Marker description	Clinical importance
Maternal anthropometric parameters			
1	Age at conception	Continuous variable expressed in number of years	Research shows that a mother’s age can significantly impact fetal development due to pregnancy related issues. This is primarily because age related changes in DNA expression can occur during critical phases of embryonic and fetal development. Mitochondria, the energy-producing organelles, are inherited solely from the mother. Unlike nuclear DNA, mitochondrial DNA lacks robust repair mechanisms, making it more susceptible to mutations as mothers age^28,29^
2	Height	Continuous variable expressed in cm	Numerical data expressed in centimetres. Numerous studies suggest a strong relationship between maternal height and birthweight^30–32^
3	Maternal weight in the first trimester	Continuous variable expressed in kg	Numerical data expressed in kilograms. Studies suggest direct associations between maternal weight, BMI, gestational weight gain, with birthweight and infant adiposity^33,34^
4	Body mass index (BMI)	Continuous variable expressed in kg/m^2^	Maternal BMI is a strong indicator of pregnancy outcomes and serves as a marker for a healthy weight^34^
5	Parity	Variables are categorised as primigravida, multigravida and encoded	Maternal parity may influence birthweight, with studies showing that newborns of primigravida mothers typically have LBW than those of multigravida mothers^35,36^
6	Type of conception	Variables are categorised as natural conception, invitro fertilisation pregnancy, ovulation induction conception, and encoded	The type of conception influences birthweight. Studies also suggest differences in developmental outcomes with assisted reproductive technology^37,38^
First-trimester markers-maternal parameters			
7	Haemoglobin (Hb)	Continuous variable expressed in g/dl	Early pregnancy hemoglobin levels were measured during the first trimester, and a meta-analysis indicates a strong correlation between these levels and pregnancy outcomes^39^
8	Glycated hemoglobin (HbA1c)	Continuous variable expressed in %	HbA1c acts as a representative of glycemic control. Poor glycemic control may lead to ketoacidosis, infection, macrosomia, dystocia, spontaneous miscarriage, and congenital anomalies^40,41^
9	Thyroid-stimulating hormone (TSH)	Continuous variable expressed in μIU/mL	Numerous studies have explored the effect of TSH on pregnancy, though the findings to date have shown variability and continue to be the subject of ongoing discussion^42,43^
First trimester anomaly scan-fetal parameters			
10	Nuchal translucency(NT)	Continuous variable expressed in mm	It is the normal accumulation of fluid present behind the neck of the fetus, usually measured between 11–13 weeks 6 days of pregnancy during FTAS^44^
11	Crown-rump length (CRL)	Continuous variable expressed in mm	It is the length from fetal head to the rump for estimating the fetal age during FTAS^44^
12	Pregnancy-Associated Plasma Protein A (PAPP-A)	Continuous variable expressed in MoM	It is a growth factor in normal fetal development and predicts the development of preeclampsia^45,46^. Fetal β-hCG and PAPP-A assessment is done in the first trimester to screen for chromosomal abnormalities such as trisomies 21 (Down’s syndrome), 18 (Edwards’ syndrome)and 13 (Patau’s syndrome)^44^
Mid trimester anomaly scan—fetal parameters			
13	Bi-parietal-diameter (BPD)	Continuous variable expressed in mm	Transverse view of the fetal head at the level of the thalami^47^
14	Head circumference (HC)	Continuous variable expressed in mm	
15	Abdominal circumference (AC)	Continuous variable expressed in mm	Transverse section of the fetal abdomen^47^
16	Femur length (FL)	Continuous variable expressed in mm	The longest axis of the ossified diaphysis of the femur bone is measured^47^
17	Estimated fetal weight (EFW)	Continuous variable expressed in grams	EFW helps in monitoring the fetal growth and development. It is calculated by the composite measurement of BPD, HC, AC, and FL^47^
Risk factors			
18	Gestational diabetes mellitus (GDM)	Variables are categorised and encoded as normoglycemic and hyperglycemic	Diabetes mellitus is significantly linked to increased birthweight and a higher likelihood of delivering infants classified as large for gestational age (LGA) or with macrosomia^48,49^
19	Hypertension (HTN)	Variables are categorised and encoded as normotensive and hypertensive	Maternal HTN has been established as a significant risk factor associated with LBW^50^
Target variable			
20	Birthweight	Expressed in grams	Classified as follows^51^ NBW : More than 2500 g LBW : Less than 2500 g VLBW : Less than 1500 g

The additional contributions of this study are as follows:

- Nineteen clinical features from early and mid-pregnancy were considered.
- Data analysis was performed using Jamovi (version: 2.6.26) and JASP team (2024) JASP (version:0.18.3) to investigate variations and patterns.
- Multiple ML models, such as Random Forest (RF), Logistic Regression (LR), Decision Tree (DT), K-Nearest Neighbors (KNN), CatBoost, LightGBM, AdaBoost, and Extreme Gradient Boosting (XGBoost) were employed to predict birthweight. These algorithms were integrated using a novel stacking architecture.
- XAI techniques, namely SHAP (Shapley Additive Explanations), LIME (Local Interpretable Model-agnostic Explanations), and Anchor, were used to enhance model interpretability.

## Materials and methods

### Dataset description

This observational prospective study was conducted between August 2022 and August 2024 at Dr. TMA Pai Hospital in Udupi and Kasturba Hospital in Manipal, under the Department of Obstetrics and Gynecology. Ethical clearance was obtained from the Institutional Ethics Committee of Kasturba Medical College and Kasturba Hospital (clearance number: IEC1:122/2022). The clinical trial was registered with the Clinical Trials Registry-India (CTRI) under the registration number CTRI/2022/08/044770. All methods were performed in accordance with relevant guidelines and regulations. Ultrasound examinations followed the protocols set by the International Society of Ultrasound in Obstetrics and Gynecology (ISUOG)^44,47^.

Only singleton pregnant women visiting for their first-trimester anomaly scan (FTAS) were recruited for the study. The sample size was determined using a single proportional formula: n = 4PQ/D^2^, based on a literature review. We identified the prevalence value as 0.667 and the margin of error (d) as 5%, with a 95% confidence interval. Consequently, the required sample size was calculated to be 356. Only 237 out of the 356 pregnant women who delivered at our institute hospitals were included for the analysis. Informed consent was obtained from all participants during recruitment. The dataset includes 19 clinical markers of these 237 pregnant women, along with the birthweights of their neonates. Maternal anthropometric parameters, such as age, height, weight, and BMI at the time of the FTAS, were collected. We also gathered information on parity, Hb level, HbA1C, thyroid-stimulating hormone (TSH), and pregnancy-associated plasma protein A (PAPP-A) from dual marker tests performed as a part of standard of care.

Ultrasound parameters, such as the crown-rump length (CRL) of the fetus, was measured to determine fetal age, and nuchal translucency (NT) thickness was assessed during the FTAS. Fetal biometry, which includes biparietal diameter (BPD), head circumference (HC), abdominal circumference (AC), femur length (FL), and estimated fetal weight (EFW), was evaluated during a routine mid-trimester anomaly scan (MTAS) performed between 18 and 24 weeks of pregnancy. Common risk factors, such as gestational diabetes mellitus (GDM) and hypertension (HTN), significantly influence pregnancy outcomes; therefore, this was documented^52^.

**Fig. 3:**
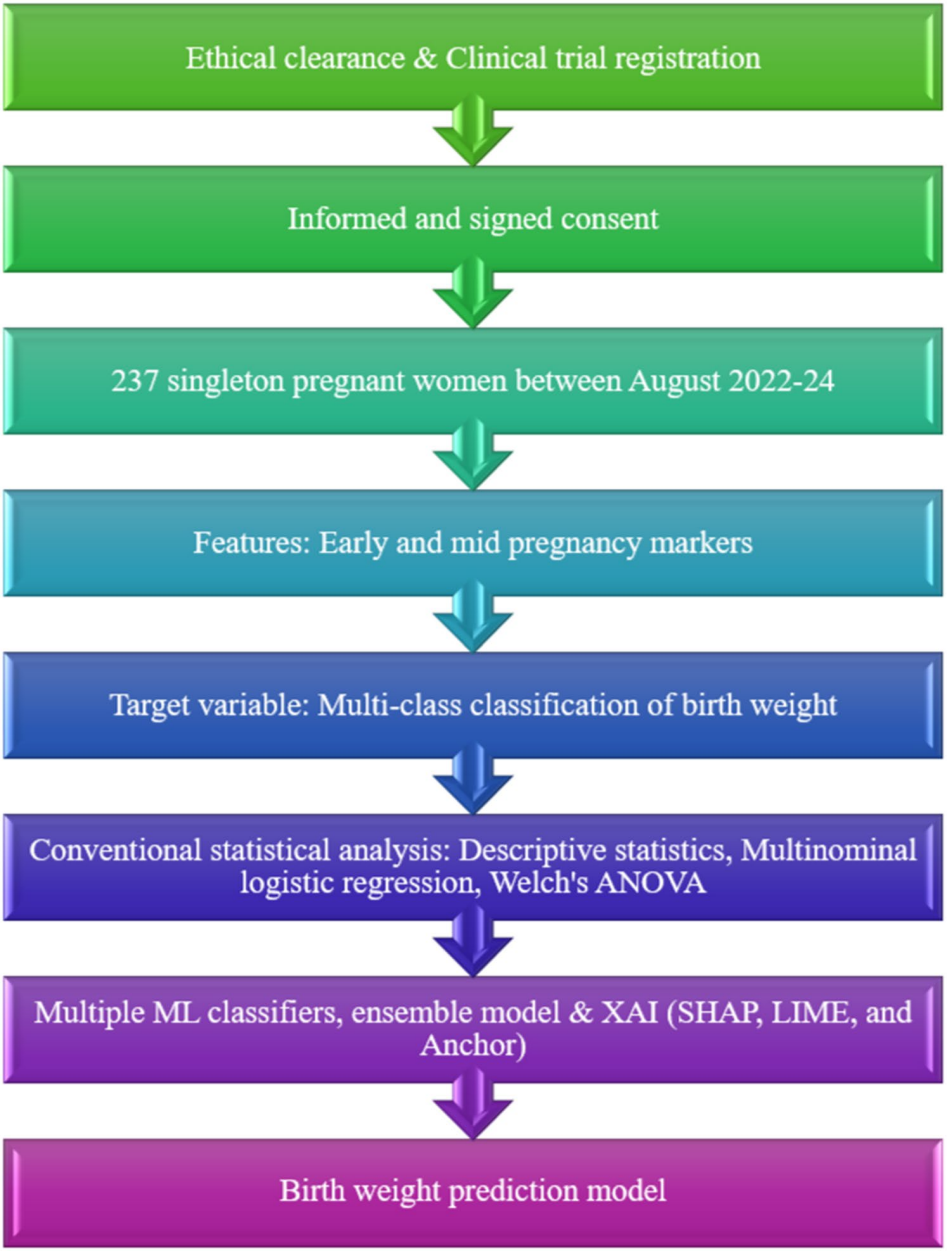
The flowchart illustrates the participant selection process for the study and its outcomes.

Our study aims to identify both modifiable and non-modifiable risk factors during early and mid-pregnancy in order to help prevent LBW. We systematically selected 19 clinically relevant features for the birthweight prediction model, which are detailed in Table 2. An experienced obstetrician reviewed all selected variables to ensure their medical significance. This review confirmed that only features essential to patient care were included. Importantly, all selected parameters are part of routine clinical practice and do not require any additional testing for this study. Figure 3 represents the flow of the study.

### Statistical analysis

Statistical analysis was conducted using Jamovi (version: 2.6.26) and JASP team (2024) JASP (version:0.18.3). The data were assessed for normality using the Shapiro–Wilk test. Normally distributed data are presented as mean ± SD, if not as medians with quartiles [M (P25, P75)]. Inferential statistical analyses were performed using Welch’s ANOVA test given the unequal sample sizes across the three groups (VLBW, LBW and NBW)^53^. Multinomial logistic regression (MLR) was done to find the association between birthweight and antenatal parameters, and Pearson’s correlation was used to identify the relationship between continuous variables. The detailed results are given in the following figures and tables. P < 0.05 was considered as statistically significant.

Table 3 provides the descriptive statistics for continuous variables and comparison of birthweight with antenatal markers via Welch’s one-way ANOVA. Statistically significant p values are represented by an asterisk.

**Table 3:** Descriptive statistics of maternal and fetal attributes and comparison of birthweight with antenatal markers via Welch’s one-way ANOVA.

Maternal features		Welch’s ANOVA test	
Attributes	Mean ± SD	F	p-value
Maternal height (cm)	155 ± 6.48	10.71	0.003*
Maternal BMI (kg/m^2^)	23.1 ± 3.97	1.54	0.258
	Median IQR		
Age (years)	29 [27, 32]	1.07	0.379
Weight (kg)	55 [47, 62]	10.72	0.003*
Hb (g/dl)	11.4 [12.3, 12.9]	0.60	0.565
HbA1C (%)	5.2 [5, 5.4]	0.83	0.458
TSH (μIU/mL )	1.73 [1.22, 2.75]	2.19	0.146
PAPP-A (MoM)	1.1 [0.81, 1.64]	4.61	0.036*
Ultrasound fetal features			
FTAS	Median IQR		
CRL (mm)	61[55.6, 65.1]	1.35	0.302
NT (mm)	1.3[1.1, 1.5]	4.01	0.036*
MTAS	Median IQR		
BPD (mm)	46 [48, 48]	0.09	0.916
HC (mm)	172 [166,178]	0.10	0.902
FL (mm)	31 [30, 33]	0.57	0.587
EFW (g)	314 [288, 353]	0.02	0.980
	Mean ± SD		
Fetal AC (mm)	147 ± 11.5	0.19	0.832

**Fig. 4:**
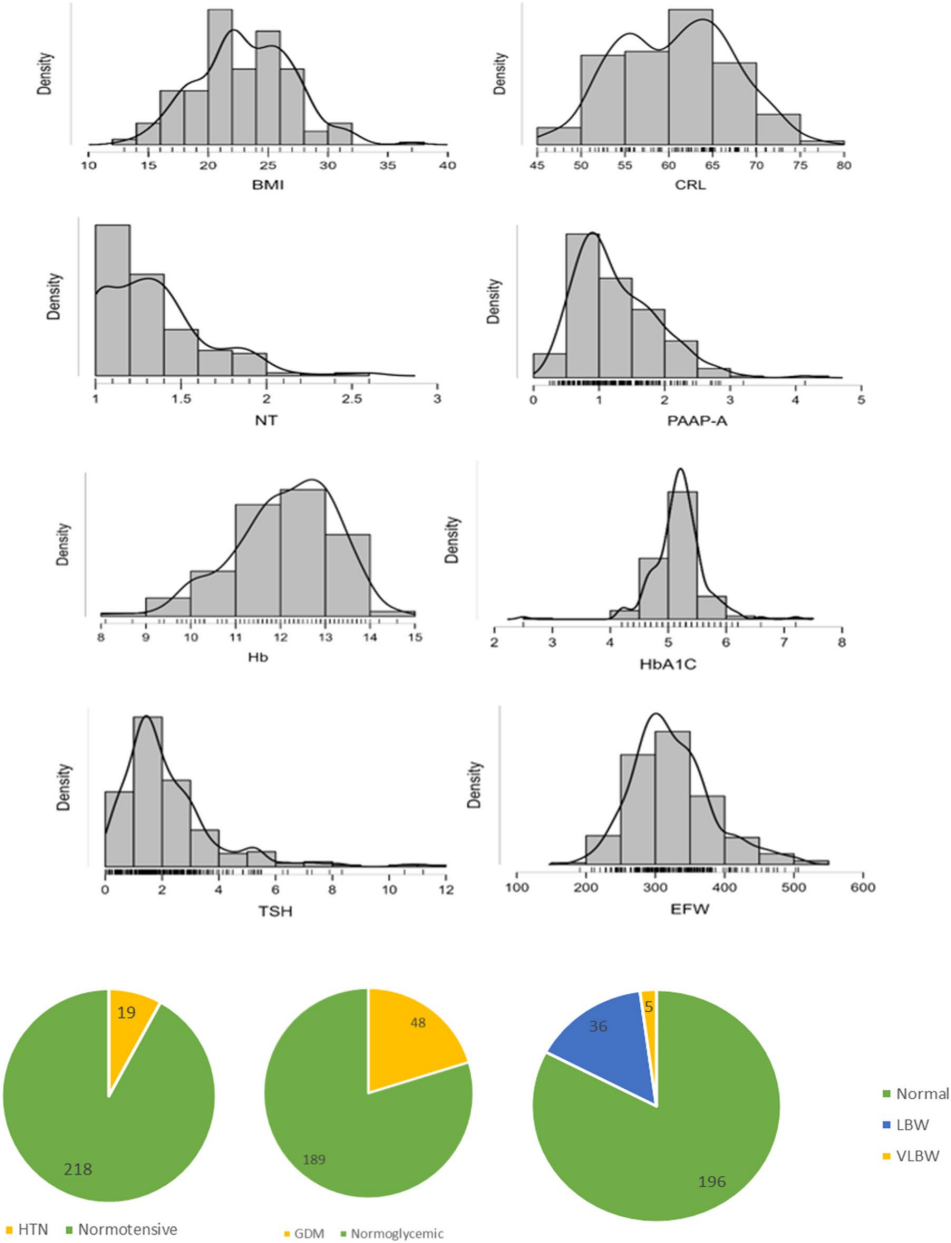
Representation of the characteristics of important attributes. The distributions of continuous variables are represented in the histogram, and categorical variables such as hypertension, gestational diabetes mellitus, and birthweight are represented in the pie diagram.

Histograms and pie charts are used to explore different patterns in the dataset, as shown in Fig. 4. The pie diagram represents the categorical data, such as GDM, HTN and birthweight.

### :

MLR analysis was done to predict the outcome variable and to find the association between birthweight and antenatal parameters. From the results, it is observed that the models have moderate fit (R^2^McF = 0.324, p = 0 0.027). These findings infer the model’s effectiveness between predictors and outcome variables. The estimates, odds ratios, p-values, and 95% confidence intervals for key predictor factors associated with antenatal features and birthweight outcomes are presented in the Table 4. The results showed that the birthweight endpoints as VLBW and LBW is associated with height, BMI, NT and FL. When NBW and VLBW were considered as references, only CRL had an association of OR 1.396, with p values of 0.036*. An odds ratio of 9.030 with a p-value (0.017) indicates a strong association between FL and birthweight. There is slightly high clinical association identified between maternal parity and reference variable OR (7.59, p = 0.153) but it is not statistically significant. The OR values of maternal weight (2.330), TSH (2.013) and Hb (1.847) had an influence on the reference variable. The OR values of CRL (1.365), PAPP-A (2.071), BPD (1.213), HC (1.017), and AC (1.104) also influenced the prediction of VLBW. Lower maternal BMI (0.108, p = 0.015) can contribute to lower birthweights. Therefore, the results suggest that maternal anthropometry, parity, first trimester parameters such as CRL and PAPP-A, and second trimester parameters such as BPD, HC, AC, FL and HTN as risk factors can affect the weight of the baby. Though the association is moderate in prediction, identifying any deviations in the above parameters may help to recognise the babies at risk of LBW. The required measures can then be taken in early and mid-pregnancy to minimise the development of VLBW and LBW as pregnancy advances.

**Table 4:** Multinomial logistic regression.

Reference	Predictor	Estimate	Odds ratio	95% confidence interval		p
				Lower	Upper	
LBW-VLBW	Age	− 0.096	0.909	0.600	1.377	0.652
	Height	− 0.565	0.569	0.325	0.994	0.048*
	Weight	0.846	2.330	0.946	5.742	0.066
	BMI	− 2.226	0.108	0.018	0.644	0.015*
	Parity: Multigravida-Primigravida	2.027	7.591	0.472	122.045	0.153
	Hb	0.614	1.847	0.459	7.439	0.388
	HbA1C	− 1.633	0.195	0.014	2.730	0.225
	TSH	0.699	2.013	0.641	6.319	0.231
	CRL	0.311	1.365	0.995	1.872	0.054
	NT	− 2.831	0.059	0.006	0.584	0.016*
	PAPP-A	0.728	2.071	0.127	33.799	0.609
	BPD	0.193	1.213	0.434	3.390	0.713
	HC	0.017	1.017	0.686	1.509	0.933
	AC	0.099	1.104	0.695	1.756	0.675
	FL	2.201	9.030	1.490	54.721	0.017*
	EFW	− 0.121	0.886	0.758	1.036	0.129
	GDM: No–Yes	− 0.398	0.672	0.032	14.309	0.799
	HTN: No–Yes	− 1.330	0.265	0.030	2.313	0.229
NBW-VLBW	Age	0.004	1.005	0.681	1.482	0.982
	Height	− 0.185	0.831	0.480	1.438	0.508
	Weight	0.600	1.823	0.744	4.468	0.189
	BMI	− 1.546	0.213	0.036	1.257	0.088
	Parity: Multigravida-Primigravida	1.324	3.759	0.262	54.004	0.330
	Hb	0.526	1.692	0.441	6.488	0.443
	HbA1C	− 1.229	0.293	0.022	3.963	0.355
	TSH	0.498	1.645	0.538	5.033	0.383
	CRL	0.333	1.396	1.023	1.904	0.036*
	NT	− 2.189	0.112	0.012	1.030	0.053
	PAPP-A	1.018	2.769	0.180	42.494	0.465
	BPD	0.012	1.012	0.375	2.733	0.981
	HC	0.115	1.122	0.764	1.647	0.559
	AC	0.092	1.097	0.697	1.725	0.690
	FL	1.631	5.109	0.878	29.725	0.069
	EFW	− 0.110	0.896	0.769	1.043	0.156
	GDM: No–Yes	− 0.707	0.493	0.026	9.237	0.636
	HTN: No–Yes	1.733	5.660	0.624	51.308	0.123

**Fig. 5:**
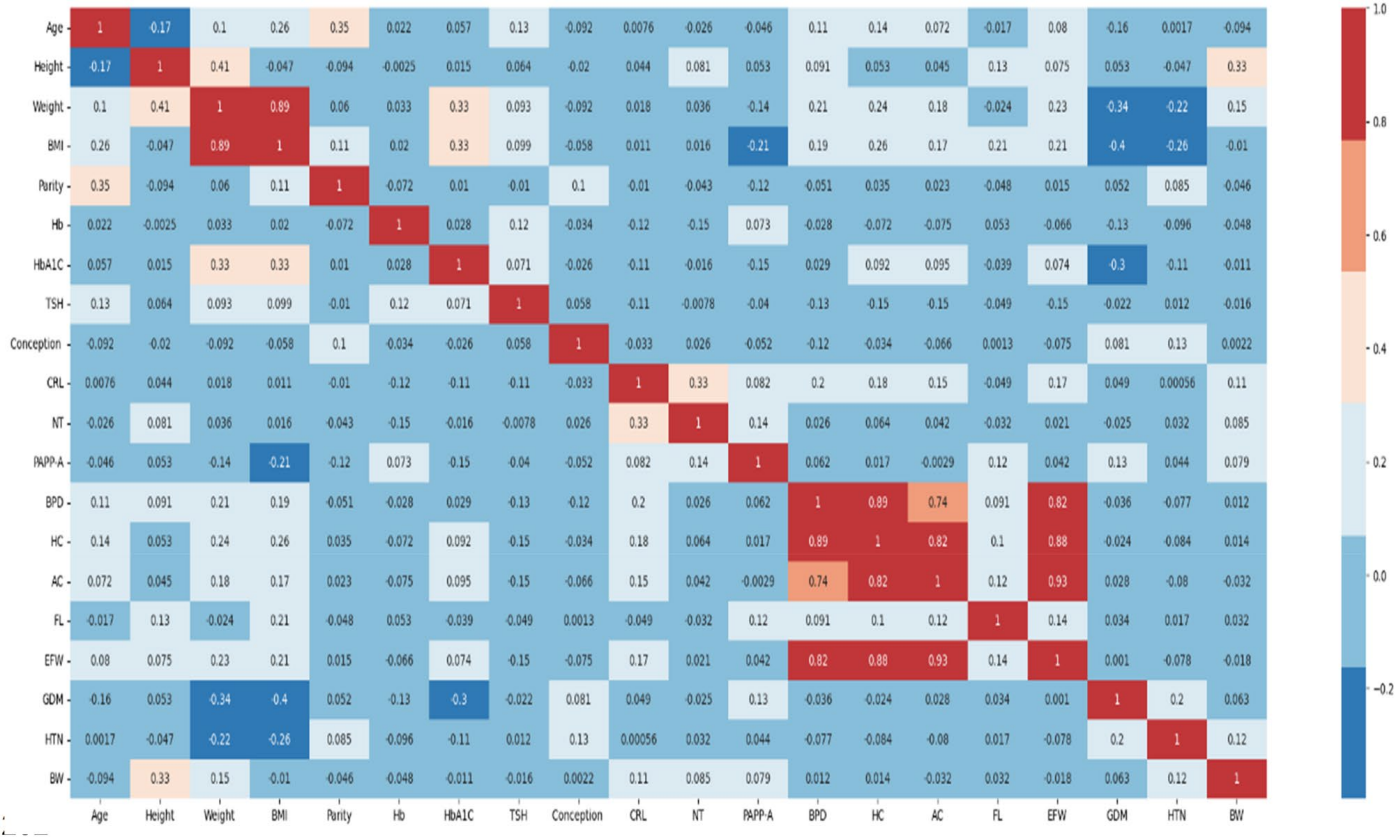
Pearson’s matrix represents the correlation between birthweight and different attributes. Maternal height, CRL, NT, PAPP-A, and HTN are correlated with birthweight (BW).

Figure 5 represents Pearson’s correlation matrix, which explains the correlation between birthweight and other variables of interest in the study. Maternal height has a positive correlation of 0.33, and first-trimester parameters such as CRL, NT, and PAPP-A had an influence on birthweight. HTN had a correlation of 0.12.

### Data preprocessing

Preprocessing the dataset is a crucial step in ML. Missing values were replaced with the respective median values to maintain consistency. Continuous features were scaled using standardization to ensure they have a mean of zero and a standard deviation of one. Categorical attributes were encoded into numerical values, as most classifiers cannot handle text inputs. Additionally, data balancing techniques were applied to address any class imbalances and improve model performance^54,55^.

Accuracy tends to decrease when there is a substantial disparity among data points. Regardless of the units used, classifiers often give more importance to features with larger values. In ML, two common methods for feature scaling are normalization and standardization. Since standardization is more robust to outliers, it was chosen for this study. After preprocessing, the dataset was split into training and testing sets using an 80:20 ratio. Notably, the dataset exhibited a significant class imbalance, with considerably fewer VLBW and LBW cases compared to NBW.

The models tend to favour majority classes, which can lead to biased conclusions when working with imbalanced data. To address this issue, we employed an oversampling method called the Synthetic Minority Over-sampling Technique (SMOTE)^54^. This technique utilizes the K-nearest neighbors algorithm to generate new synthetic samples, proving particularly effective for borderline instances. In this study, we avoided under-sampling to ensure that we did not miss any significant trends or patterns in the data. We also chose not to balance the test data to maintain data integrity.

Feature selection is crucial for reducing redundant data and minimizing noise. However, it is important to note that SMOTE can introduce noise while creating synthetic data and handling missing values, which may affect the model’s accuracy. To tackle these challenges, we implemented several ML models as well as stacking ensemble models^56,57^. Additionally, we used XAI techniques such as SHAP, LIME, and anchor to enhance the model’s transparency and performance.

**Fig. 6:**
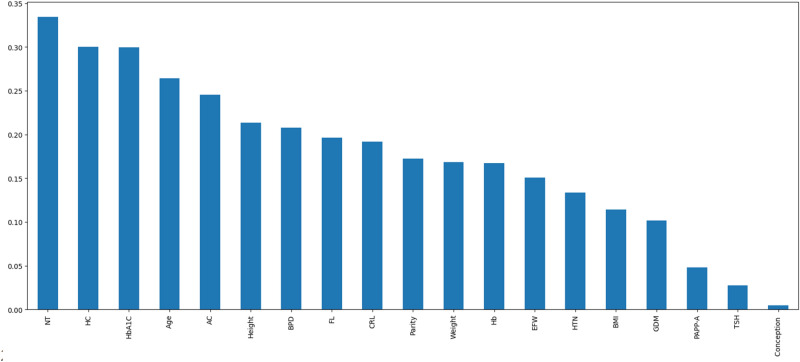
Representation of mutual information. It represents the importance of different variables according to the importance in descending order. NT, HC, HbA1c, age, and AC are considered important features and occupy the initial portion from left to right.

The next step was to address the curse of dimensionality, reduce overfitting by enhancing generalization, and shorten the training period. A mutual information curve was used to identify the features that significantly contributed to the model. The highest importance was observed at the beginning of the curve^58^. Figure 6 illustrates a bar diagram based on this mutual representation. The analysis showed that the most significant contributors to the model were NT, HC, HbA1C, age, AC, height, and BPD. In contrast, the type of conception, TSH, PAPP-A, GDM, and HTN ranked lower in terms of priority. This may be because the pregnant women received treatment during pregnancy, and these factors are modifiable; thus, the birth outcomes improved with treatment.

### Machine learning optimisation

**Fig. 7:**
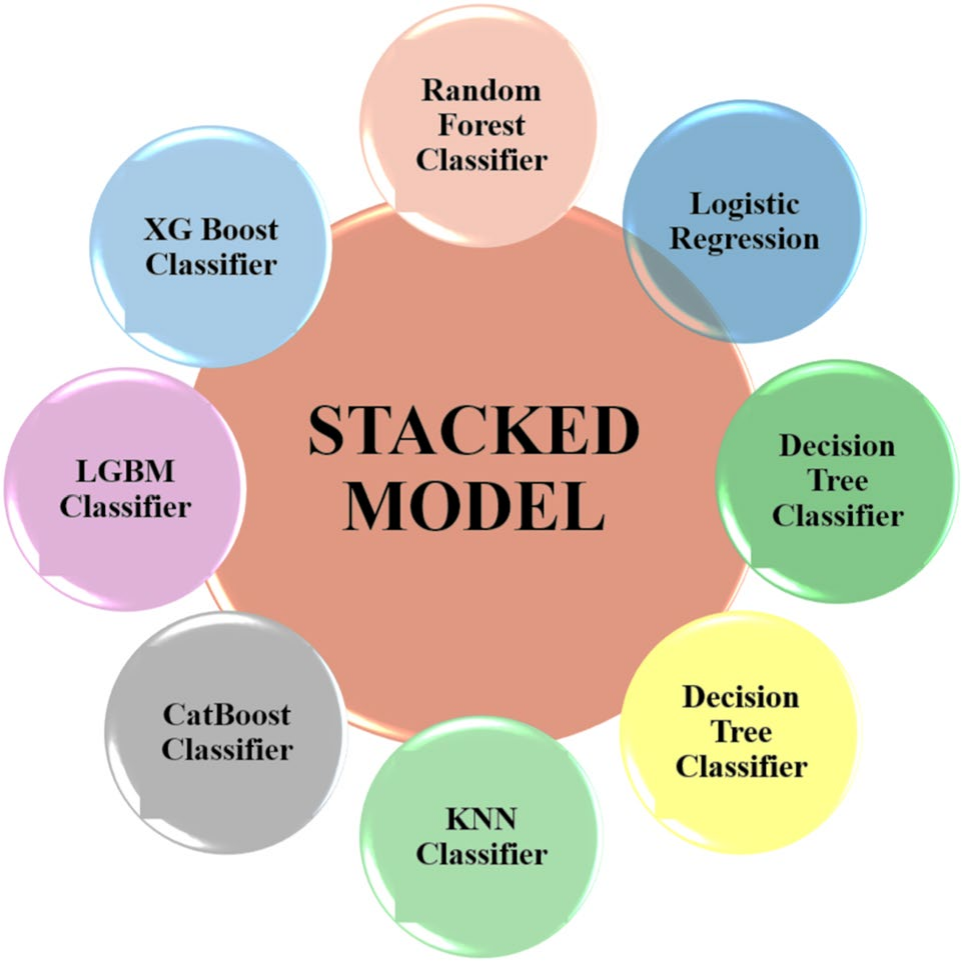
Eight different types of ML classifiers applied in the study and a stacked model is developed.

ML employs algorithms that systematically analyze and model complex relationships within data. By leveraging previous data as input, ML enables algorithms to predict outcomes with high accuracy. In this study, several ML classifiers including RF, LR, DT, KNN, AdaBoost, CatBoost, LGBM, and XGBoost were utilized. These classifiers were selected for their proven effectiveness in creating robust predictive models, especially when dealing with real-world medical datasets. They are capable of handling categorical and numerical data, managing missing values, and are easy to implement, making them well-suited for medical applications^59–64^. Furthermore, a stacked ensemble model was utilized, combining eight different models to enhance the accuracy of each individual classifier. This stacking strategy aggregates predictions from various baseline models, leading to improved overall predictive performance. Figure 7 shows the ML methodology used in this study.

To train our predictive model, we used a combination of preprocessing and validation techniques to enhance performance and ensure robustness. This included data normalization, hyperparameter tuning via grid search, cross-validation, and class balancing using SMOTE. Normalisation of data helps reduce the influence of outliers and ensures that the model treats all features equally. As a result, the model can more effectively detect meaningful patterns in the data, which contributes to improved prediction accuracy and overall model performance^65^. The effectiveness of a classifier is influenced by the chosen hyperparameters and their tuning. The model performs well on a validation set if the hyperparameters are fine-tuned before the learning process begins. Hyperparameter values to identify the combination of features that creates the best model performance. To prevent overfitting and to assess the generalization ability of predictive models cross-validation data resampling method was done^66,67^. Grid search and tenfold cross validation was done as hyperparametric optimisation. By utilizing the grid search method, the trained model is ensured to capture various patterns within the dataset. This approach essentially creates a grid that includes every possible combination of the specified hyperparameter values. Each model’s performance is evaluated based on a scoring system, and ultimately, the model that yields the best results is selected^68^. Cross-validation was utilized in the early stages of model evaluation to assess performance and reduce the risk of overfitting. We focused particularly on precision and recall, as these metrics are crucial for minimizing false positives and false negatives. The ML framework involved dividing the dataset into multiple subsets, or "folds." The model was trained on all but one-fold and validated on the remaining fold. This process was repeated, allowing each fold to serve as the validation set once. The results were then averaged to provide a more robust estimate of model performance. We implemented a tenfold cross-validation approach, which is widely used in ML evaluation. In this method, the dataset is divided into ten equal parts. Each part is used as a validation set once, while the other nine parts are employed for training. This iterative strategy ensures that the model’s predictive performance is tested on unseen data, enabling an evaluation of its generalization capability. By analyzing performance metrics across the folds, we assessed whether the model was overfitting, underfitting, or generalizing appropriately^69,70^.

To reduce the bias in imbalanced data of VLBW, LBW and NBW, we applied SMOTE, which generates new synthetic examples for the minority class by interpolating between existing minority. This approach helps expand the decision boundary of the minority class, reducing overfitting and improving generalizability^54^. In this study, we selected various classification and loss metrics to evaluate the models. The metrics included precision, recall, accuracy, the F1 score, Matthew’s correlation coefficient, Jaccard score, and Hamming loss.

**Fig. 8:**
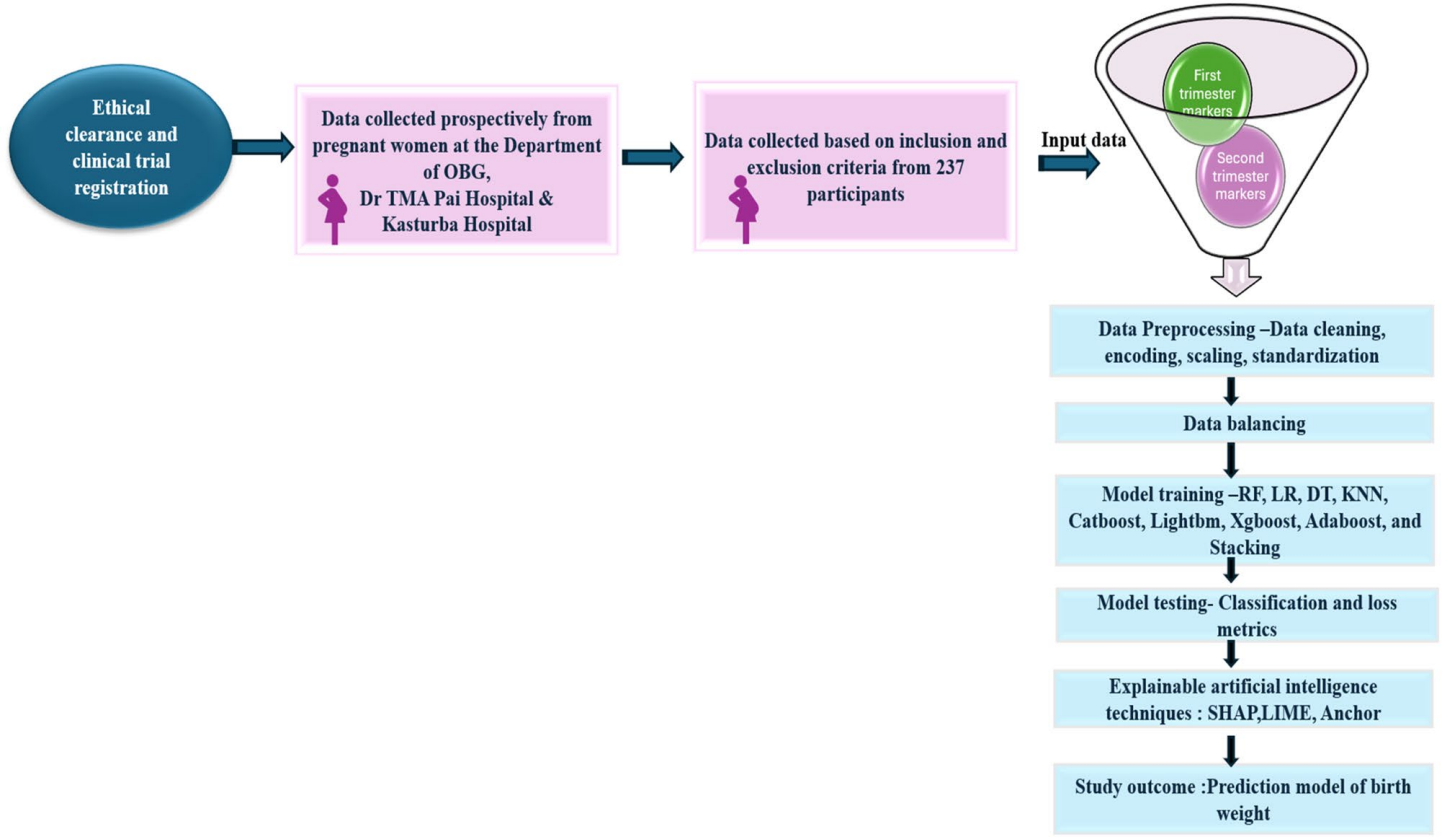
The flow chart represents the study procedure from ethical clearance to the study outcome in the development of birthweight prediction model.

XAI techniques, namely SHAP, LIME, and Anchors, were utilized in the study^71,72^. The results of the study were explained through three XAI (SHAP, LIME, and Anchor) approaches after training and testing the ML models. An elaborate ML method called SHAP is a sophisticated ML method that explains how each feature contributes to a prediction by assigning a relevance value to it. This model-agnostic tool is highly versatile, as it can be applied to any ML model. SHAP provides both local and global explanations, offering detailed insights into individual predictions as well as the overall behavior of the model. By accurately representing variations in the model’s predictions through SHAP values, consistency and reliability are ensured. Additionally, SHAP efficiently sets the corresponding values of missing features to zero. This helps identify the most significant features in a model and clarifies how each feature influences the prediction outcome. LIME works particularly well with smaller datasets and delivers insights into individual predictions that are easier to comprehend. It is versatile across diverse forms of data because of its model-agnostic nature, which enables its use in different models. Anchors explain significant features through a set of ‘rules’ and ‘conditions’. Two parameters are used to measure each anchor (condition): precision and coverage. Precision determines the accuracy of explanations. Coverage defines the number of instances that are predicted using the same conditions, which aids in understanding the model’s prediction. This adaptability improves trust in AI-driven diagnostic procedures by helping patients and clinicians acquire confidence in the diagnostic outcomes generated by AI^73^. These XAI algorithms offer insights in the form of graphs and tables, making the results simple for ML users. Figure 8 represents the flow of research from ethical clearance to the development of the prediction model.

## Results

This study provides a detailed analysis of 237 pregnant women, utilizing early and mid-pregnancy antenatal parameters to predict birthweight. We applied conventional statistical analysis, various ML classifiers, and XAI techniques to develop reliable and optimal clinical models.

In this section, we discuss the performance of the classifiers used. Table 5 summarizes the results from various models. The AdaBoost model achieved the highest performance, with a maximum accuracy of 77%, the highest precision of 73%, a recall value of 77%, and an F1 score of 72%. The stacked model reached an accuracy of 75%. The Jaccard score indicated a high degree of overlap in the outputs of the two sets, with the AdaBoost classifier model yielding the highest score of 0.61. Additionally, the maximum Matthews correlation coefficient (MCC) obtained through the AdaBoost classifier model was 0.33, indicating a significant correlation between the predicted and actual outputs.

**Table 5:** Results obtained by the various models.

Classifiers	Accuracy	Precision	Recall	F1 score	Hamming loss	Jaccord score	Mathews correlation coefficient
1. RF	0.71	0.53	0.71	0.60	0.29	0.51	− 0.08
2. LR	0.58	0.65	0.58	0.60	0.41	0.44	0.12
3. DT	0.58	0.62	0.58	0.60	0.41	0.42	0.05
4. KNN	0.65	0.61	0.65	0.62	0.35	0.49	0.01
5. AdaBoost	0.77	0.73	0.77	0.72	0.22	0.61	0.33
6. CatBoost	0.73	0.66	0.73	0.65	0.27	0.54	0.11
7. LGBM	0.73	0.66	0.73	0.65	0.27	0.54	0.11
8. XG Boost	0.71	0.62	0.71	0.63	0.29	0.52	0.04
9. Stacked model	0.75	0.72	0.75	0.69	0.25	0.57	0.23

Results of the performance measures of birthweight prediction of the models. RF, random forest; LR, logistic regression; DT, decision tree; LGBM, light gradient boosting machine; XG, boost extreme gradient boosting. AdaBoost showed high accuracy and precision, recall the least hamming loss, and a high Jaccord score. Mathew’s correlation is also given with each model.

**Fig. 9:**
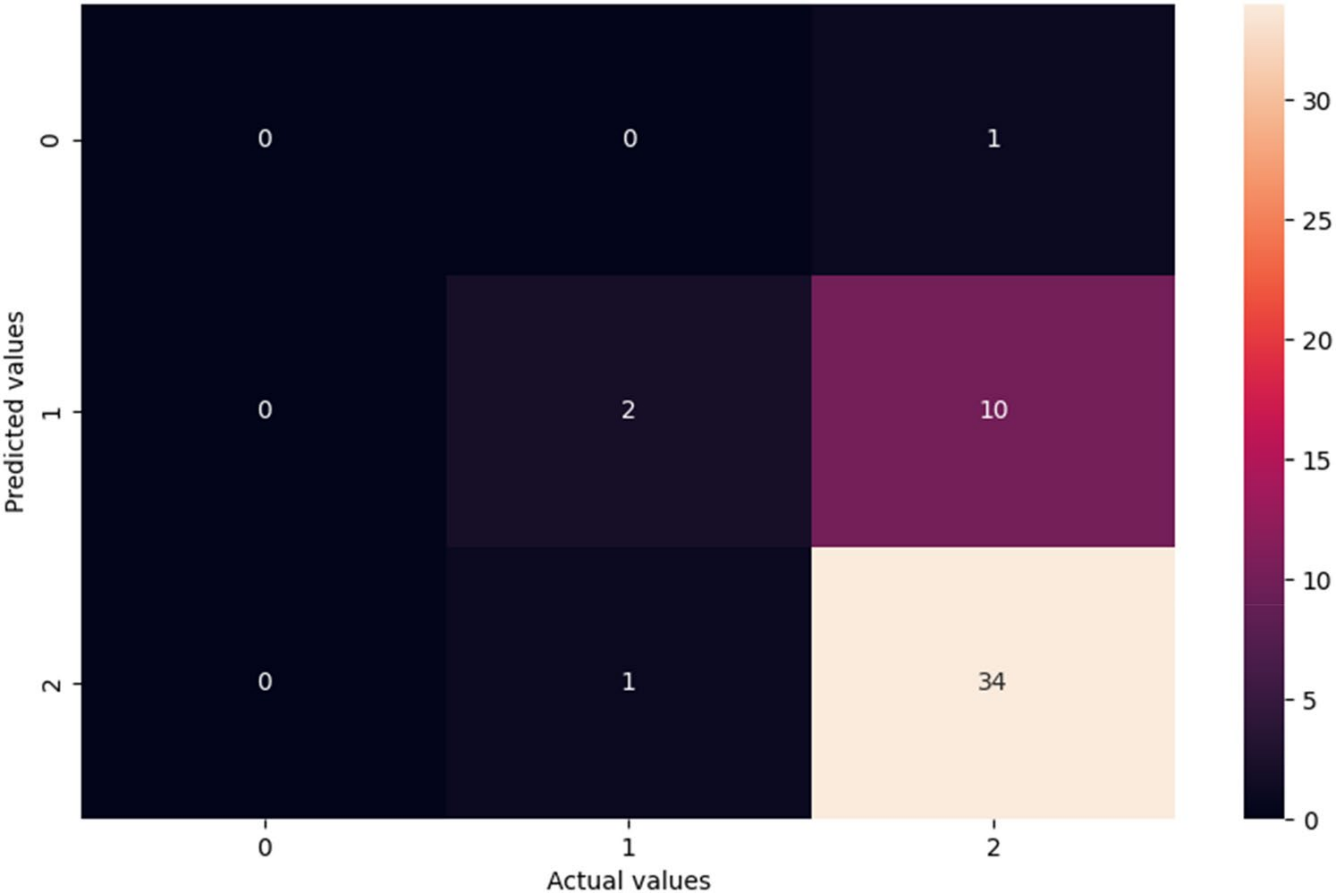
Confusion matrix of multi class obtained for the stacked model.

Figure 9 shows the confusion matrix generated for the stacked model applied to the dataset. The performance of the classification algorithm is summed and visualised using a confusion matrix. The confusion matrix describes the performance of the classification method. Generally, the outputs are classified as true positives, true negatives, false positives, or false negatives. It can be used to calculate a variety of model performance metrics. The model showed good overall prediction accuracy for NBW. Notably, cases of NBW were identified much more frequently than those of LBW. However, the prediction performance for VLBW instances was significantly lower, most likely due to a lack of training data for this category.

**Fig. 10:**
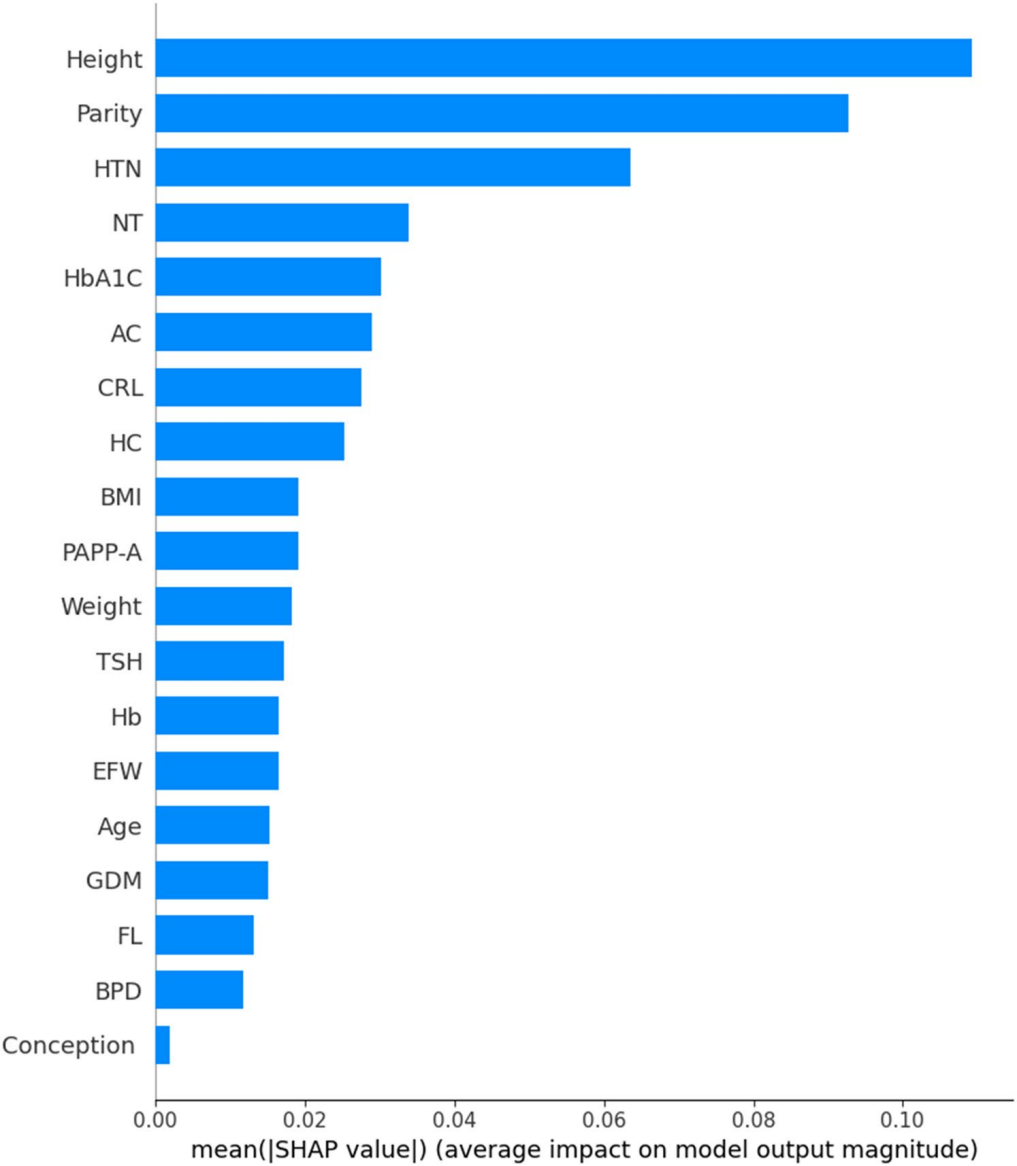
SHAP plot indicating different attributes arranged in descending order. Important attributes such as height, parity, HTN, NT, HbA1C occupy the initial levels.

Figure 10 illustrates the ranked importance of the features determined using the SHAP model. The key features, in descending order of significance, include height, parity, HTN, NT, and HbA1C. In contrast, features such as conception type, BPD, FL, GDM, age, EFW, Hb were deemed less important by the model.

**Fig. 11:**
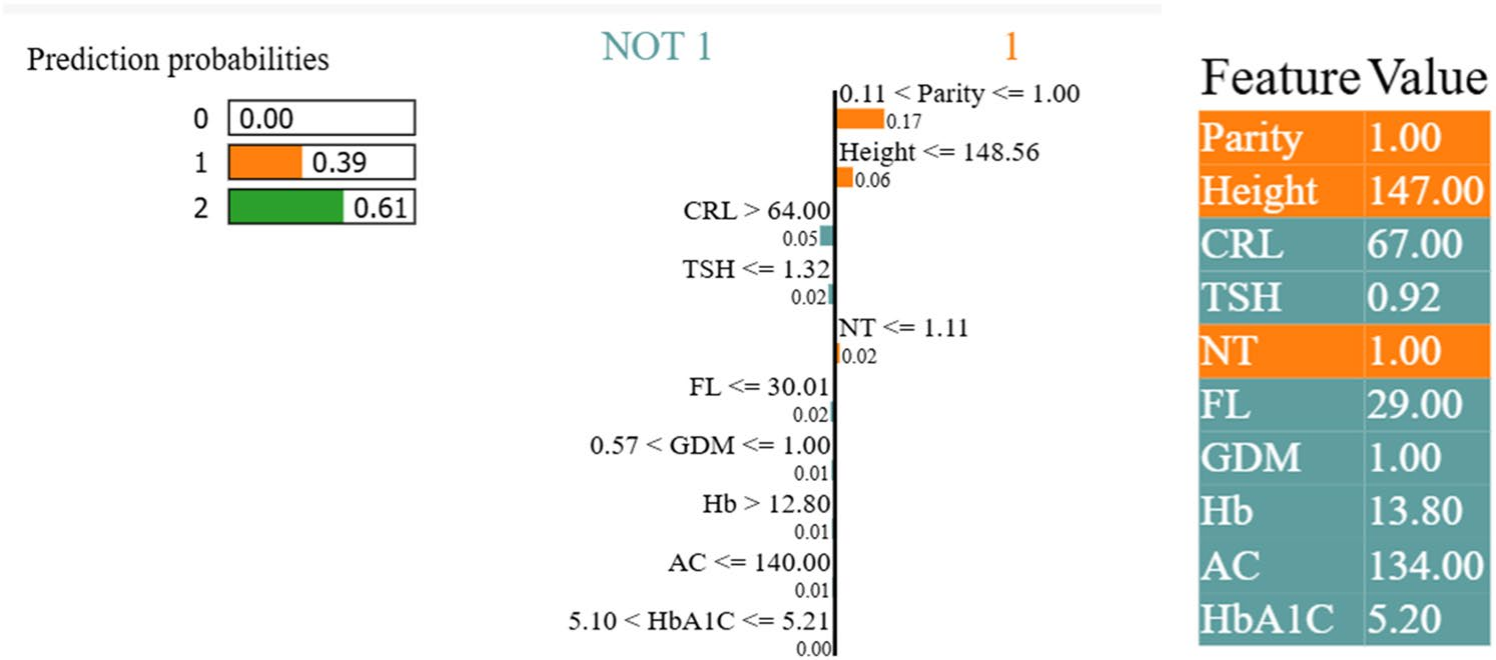
Model explainability using LIME. Parity and height influence the outcome.

Figure 11 shows the LIME model for the prediction of birthweight. The LIME model identified parity and height as strong predictors. Additionally, the CRL and TSH are recognised as contributing factors in determining birthweight. Sixty-one per cent of the NBW and 39% of the LBW babies were differentiated. The prediction of VLBW was negligible because the dataset contained minimal VLBW babies.

The anchors for different birthweights are listed in Table 6. Similarly, parity, HC, BMI, height, CRL, and HbA1c are considered significant anchor conditions for determining birthweight.

**Table 6:** Model interpretability with anchor.

Instance	Patient prediction	Anchor condition	Precision	Coverage
1	Very low weight	Parity < = 0.11 AND BMI < = 24.20	0.65	0.30
2	Very low weight	Parity < = 0.00 AND BMI < = 24.20	0.63	0.27
3	Low weight	Parity < = 0.11 AND Height > 153.22	0.73	0.26
4	Low weight	HC < = 172.00 AND Height < = 148.56	0.77	0.16
5	Low weight	Parity < = 0.00 AND CRL > 64.00	0.74	0.12
6	Low weight	Parity < = 0.00 AND HC > 175.00	0.76	0.11
7	Low weight	Height > 157.09 AND HbA1C < = 5.21	0.68	0.12
8	Normal	Height > 153.22 AND HbA1C < = 5.10	0.69	0.14
9	Normal	Parity < = 0.00 AND Height > 153.22	0.74	0.24
10	Normal	Height > 157.09 AND CRL > 64.00	0.80	0.06

## Discussion

AI algorithms show promising potential in advancing medical research because of their ability to process and analyze large-scale datasets and facilitate the development of predictive tools^74^. In recent years, researchers have adopted a multidisciplinary strategy to integrate computer science, engineering, and maternal–fetal medicine into obstetrical care. ML has demonstrated encouraging results in improving predictive models for various obstetric outcomes, such as GDM, preeclampsia, preterm birth, fetal ultrasound image analysis, gestational weight gain, early diagnosis of fetal alcohol spectrum disorder, fetal hypoxia detection, and birthweight^75,76^. Among these applications, predicting birthweight is particularly important for public health, especially in low-resource settings were estimating gestational age accurately can be difficult. LBW is a crucial indicator of neonatal health, which further emphasizes the need for reliable prediction methods^77^. Our study employed conventional statistical methods and multiple ML algorithms to predict birthweight, supporting more effective treatment planning beginning in early pregnancy to help avert progression to LBW newborns. To enhance model transparency, we incorporated three XAI approaches. These explainers allow doctors and other medical professionals to quickly identify variations in key markers.

Table 7 illustrates the significance of the various features assessed via conventional statistical methods and ML techniques. Maternal height, NT thickness, parity, CRL, HbA1C, HTN and PAPP-A are considered important attributes of birthweight according to our model developed via statistical tools, mutual information graphs and XAI techniques. The differences in feature importance arise primarily because statistical methods and ML algorithms operate on different principles. Statistical models rely on predefined assumptions to infer relationships between variables, while ML algorithms focus on identifying complex patterns in data to enhance predictive accuracy. Despite the variation in feature rankings across models, it is noteworthy that the features consistently identified as important possess established clinical relevance. All variables included in this study were carefully selected based on evidence-backed international guidelines and the expert consensus of experienced clinicians, ensuring both statistical rigor and clinical applicability.

**Table 7:** Important features for predicting birthweight with different statistical and ML algorithms.

Welch’s ANOVA	Multinominal logistic regression	Pearson’s correlation matrix	Mutual information	SHAP	LIME	Anchor
NT PAPP-A Height	CRL PAPP-A BPD HC AC FL HTN	Height CRL NT PAPP-A HTN	NT HC HbA1c Age AC	Height Parity HTN NT HbA1C	Parity Height CRL	Parity BMI Height CRL HbA1c

According to our model, important predictors of birthweight include maternal height, NT thickness, parity, age, BMI, CRL, HbA1C, HTN, and PAPP-A.

### Maternal height

Our findings indicate that maternal heights of less than or equal to 148.56 cm, combined with fetal head circumferences less than 172 mm measured during MTAS, are associated with high instances of LBW. This aligns with existing studies that suggest maternal height significantly influences birthweight. Conversely, maternal heights exceeding 153.22 cm, along with HbA1c levels of less than or equal to 5.10, are linked to a higher incidence of NBW. Research indicates that taller mothers tend to have heavier infants, with this trend varying based on ethnicity and genetic factors. Specifically, studies involving Malaysian ethnic groups show that newborn birthweight increases with maternal height, with an estimated increase of 7.08 g in birthweight for every 1 cm increase in maternal height. Data from European countries suggest that this increase can range from 15 to 17 g per centimetre of maternal height. A study conducted on 8541 mothers and their children from various ethnic backgrounds in Rotterdam, the Netherlands, examined the relationship between maternal body measurements and fetal weight throughout pregnancy, as well as birth outcomes. The findings revealed that maternal BMI starts to impact fetal growth from the middle of pregnancy onwards. Additionally, factors such as maternal height, pre-pregnancy BMI, and weight gain during pregnancy were associated with the likelihood of having babies who are either smaller or larger than expected for their gestational age. However, the exact biological mechanisms underlying these associations are not yet fully understood. Various studies around the globe also support the influence of maternal height on birthweight^78–91^.

BMI is considered one of the factors associated with birthweight according to anchor. According to the anchor, a maternal BMI <  = 24.20 kg/m^2^ is associated with LBW, as BMI is a composite value of height and weight. The mother’s weight in the current study was measured during the first trimester. Retnakaran et al. found that weight gain before 18 weeks of gestation, particularly from pre-pregnancy to 14 weeks and from 14 to 18 weeks, was significantly associated with higher birthweight. In contrast, weight gain after 18 weeks did not show a notable impact, emphasizing that early pregnancy is a critical period for interventions aimed at optimizing birth outcomes. These findings demonstrate that maternal weight status in early pregnancy is a significant predictor of birthweight^92^. A study conducted in India by Mohapatra et al. supports these findings, showing a strong relationship between early pregnancy BMI and LBW. A low BMI during the early stages of pregnancy has been linked to LBW and preterm birth. Furthermore, the socioeconomic status of pregnant women may influence their weight gain by affecting access to healthy food, nutrition, antenatal care, and health supplements^93,94^.

**NT:** NT thickness has been identified as a significant factor affecting birthweight. This finding is consistent with research by Hackmon et al., who established a strong correlation between birthweight ratios and fetal measurements, including CRL, BPD, and NT. These results suggest that early fetal biometric measurements may reflect physiological variations in birthweight^95^. NT thickness greater than 3 mm however requires further evaluation at specialized centers due to the increased risk of chromosomal and structural anomalies^96^. Moreover, Kalem et al. found a link between higher maternal age and elevated NT multiples of the median (MoM) with an increased risk of neonatal intensive care unit admission^97^.

### CRL

Menstrual history, precisely the length and regularity of the menstrual cycle, plays a role in dating pregnancy in the first trimester. The growth of the fetus is linear, with less standard deviation between 9 and 13 weeks of pregnancy, which is determined by the CRL. If a discrepancy arises between the observed value with fetal biometry through USG and the menstrual cycle, the dating is revised as the corrected estimated delivery date (C-EDD). These findings lay the foundation for tracking fetal growth as pregnancy advances. With this type of calculation of gestational age, over-estimation or underestimation is reduced. The misclassification of SGA infants, large babies, early inductions, or post-term deliveries is minimized^98–101^. The XAI technique called anchor suggested that a CRL greater than 64 mm and a maternal height greater than 157.09 cm resulted in NBW.

**Parity:** Parity of the mother was determined to be one of the contributing factors for birthweight in our study results. All the XAI techniques, SHAP, LIME, and anchor, consider parity to be one of the factors influencing birthweight. This finding emphasizes the importance of considering maternal parity in clinical decisions regarding birthweight. A meta-analysis has demonstrated that nulligravida mothers tend to have infants that weigh 280 g less than those of multigravida mothers. Physiological and anatomical changes that occur during the first pregnancy may impact subsequent pregnancies. Increases in uterine size, uteroplacental circulation and nutritional status are potential factors that benefit mothers with multiple pregnancies. Consequently, these elements help reduce the likelihood of LBW^36,102–107^. However, as age advances, the risk of anaemia, premature birth, and macrosomia increases^108,109^.

**PAPP-A:** PAPP-A is considered a contributing factor in MLR, Welch’s ANOVA, and Pearson’s correlation. However, XAI did not consider it an important variable. PAPP-A is estimated during aneuploidy screening in the first trimester. It also acts as a predictor of preeclampsia and has an influential role in birthweight. Low levels of PAPP-A are also indicators of placental dysfunction^110,111^. Consequently, any change in placental blood flow can lead to a decreased nutrient supply to the developing fetus, resulting in LBW.

**HbA1c: **HbA1c is a critical feature of mutual information, SHAP and anchor. Zhao et al., who reported that mothers with elevated blood sugar levels during pregnancy had a greater risk of macrosomic infants^112^.

**HTN:** HTN was identified as an important factor by MLR, Pearson’s correlation and SHAP. Gestational HTN and preeclampsia cause endothelial dysfunction, which subsequently decreases uteroplacental blood perfusion. This underlies the pathophysiological basis of LBW and preterm birth^113,114^.

**Maternal age: **Maternal age is identified as one of the factors measured by mutual information; however, age and birthweight are negatively correlated, with a Pearson correlation coefficient of − 0.094. Research indicates that there are increased risks of pregnancy complications, such as stillbirth, preterm birth, and intrauterine growth restriction, associated with maternal age of 19 years or younger, as well as with advanced maternal age of 35 years or older. Additionally, these risks are influenced by various factors, including socioeconomic status, educational attainment, parity, and the tendency for well-qualified women to delay pregnancy in order to pursue their careers^115–122^.

### Impact of treatment on model predictions

Pregnant women were recruited for the study between 11 and 13 weeks + 6 days of gestation. The lower gestational age limit of 11 weeks was chosen to ensure the inclusion of viable pregnancies, as most pregnancy losses between 6 and 10 weeks occur due to fetal chromosomal abnormalities, including trisomies, monosomies, and polyploidy^123,124^. Pregnancies complicated by lethal anomalies or intrauterine fetal demise were excluded during follow-up. It is well established that conditions such as GDM and HTN are linked to adverse pregnancy outcomes. Therefore, pregnant women identified as high risk, those with a history of LBW, preterm birth, HTN, and GDM received appropriate clinical intervention. Low-dose aspirin for HTN, dietary counselling, metformin, or insulin for GDM were prescribed when indicated. In our prospective study, we found that early diagnosis and timely treatment of these conditions likely contributed to a reduction in complications such as VLBW, LBW even in high-risk pregnancies.

The stacked model developed in this study achieved an accuracy of 75%, while the Adaboost model reached an accuracy of 77%. It is important to interpret these performance metrics within the context of real-world clinical data. In practice, when pregnant women are identified with risk factors, they receive prompt treatment, which often helps mitigate adverse outcomes such as LBW or preterm delivery. Consequently, the model’s predictive power may be underestimated because timely interventions can reduce the occurrence of complications that the model aims to predict.

We suggest that the observed accuracy reflects both the model’s predictive capabilities and the effectiveness of clinical interventions. Untreated populations might exhibit higher model accuracy due to a greater frequency of adverse outcomes; however, such a situation would be unethical. In clinical settings where treatment is standard, achieving very high accuracy is inherently challenging. While this success in prevention is clinically desirable, it can also lead to an underestimation of the model’s sensitivity or accuracy. Nevertheless, our model employs standard point-of-care variables, which enhances its real-time application with early and mid-pregnancy markers. Additionally, by excluding rare or niche variables, we improve the model’s generalizability and applicability across broader populations. This may reflect clinical practice patterns rather than represent a true limitation.

From a healthcare perspective, the goal is not only prediction but also prevention. Therefore, the reported accuracy of 75% signifies not just the model’s predictive power but also the positive impact of real-time medical interventions, reinforcing the model’s practical value in supporting clinical decision-making. Table 7 indicates the features such as maternal height, NT thickness, parity, age, early pregnancy BMI, CRL, HbA1C, PAPP-A, HTN as the strong predictors of LBW. In obstetrics, the inverted pyramid theory suggests that early detection and prioritization of high-risk fetuses can significantly reduce unfavourable postnatal outcomes^125^. This approach emphasizes the importance of timely referrals, cost-effectiveness, and efficient resource allocation. Our proposed model aligns with this principle by assisting clinicians in the early identification of LBW pregnancies using early pregnancy markers, enabling proactive interventions to improve neonatal outcomes.

Numerous machine learning studies have aimed to predict fetal birthweight, predominantly relying on sociodemographic variables. However, there remains a significant gap in the use of clinical data from early and mid-pregnancy within these models. Table 8 presents various ML techniques that have been previously employed in this area. To our knowledge, no published studies have utilized three distinct XAI methods to predict birthweight using real clinical data from early and mid-pregnancy. This makes our approach innovative and underscores the contribution of this study to the existing body of literature.

**Table 8:** Summary of different models for birthweight prediction.

Reference	Dataset	Learning methods used	Accuracy obtained	Critical predictors	XAI method
Bekele, W.T^126^	Sociodemographic	LR, DT,NB, KNN, RF, SVM (Support Vector Machine), GB, and XGB	RF Accuracy:91.60% Sensitivity 91.60%	Gender Marriage to birth interval Mother’s occupation Mother’s age	None
Arayeshgari M et al.^127^	Maternal and neonatal demographic characteristics	DT, RF, ANN, SVM and LR	LR Sensitivity 74% Specificity 89% Accuracy 88%	Gestational age Number of abortions Parity Consanguinity Maternal age at delivery Neonatal sex	None
Islam Pollob et al.^128^	Anthropometric, Socio demographic and pregnancy history	LR and DT	LR Sensitivity 99.5% Specificity 17.7% AUC 0.59	Region Education Wealth Height Twin pregnancy	None
Khan W et al.^129^	Arab pregnant women	Multiple ML models	RF algorithm with an MAE value of 294.53	Diabetes Gestational age HTN	None
Hussain et al.^130^	Indian pregnant women- certain features from conception to birth	Gaussian NB, RF	RF outperforms Gaussian Naïve Bayes	Physical and mental health of the mother influences the fetus	None
Alabbad D.A et al.^131^	KFUH and IEEE datasets	SVM, DT, RF, ET (extra trees), GaussianNB, XGBoost, AdaBoost, and LGBM	ET accuracy 98% (KFUH dataset) RF accuracy 96% (IEEE dataset)	Age Weight Height Gender	SHAP
Dola S et al.^132^	Anthropometric, socioeconomic, maternal, and paternal factors	LR, RF, XGBoost, conditional inference tree, and attention mechanism	XGBoost showed the highest performance	Maternal height Prepregnancy Weight Weight gain during pregnancy Parental ethnicity	Partial dependence plots (PDP), SHAP
Liu Q et al^133^	ML to predict fetal macrosomia	RF, KNN, AdaBoost, SVM, naïve Bayes, and LR	LR and ensemble model demonstrated good performance	Maternal and fetal parameters	SHAP
Current study	Birthweight prediction using early and mid-pregnancy antenatal markers	RF, LR, DT, KNN, AdaBoost, CatBoost, LGBM, XGBoost and ensembled model	AdaBoost	Maternal height NT Parity CRL HbA1C PAPP-A	SHAP, LIME, Anchor

### Strength and limitation

A major strength of our study is the use of real-world clinical data that is routinely collected during antenatal care. This enhances the model’s applicability and relevance to everyday clinical practice. Our AI-driven model successfully identified key predictors of LBW, including maternal height, NT thickness, parity, age, CRL, HbA1c, and BMI from early and mid-pregnancy. These variables are standard components of antenatal screening and can be accessed even in low-resource settings, increasing the model’s utility and scalability. The model not only aids in birthweight predictions but also supports timely clinical decision-making and early interventions. Importantly, the integration of XAI methods enhances the model’s transparency and interpretability, fostering trust among clinicians and promoting its adoption in clinical workflows. The model provides insights into high-risk pregnancies that may result in LBW or VLBW, allowing healthcare teams to proactively plan medical management strategies. This can significantly reduce the financial, psychological, and emotional burden on families and alleviate stress among healthcare professionals. Our work lays a foundation for further research not only in prediction but also in prevention strategies for LBW. Early identification of modifiable risk factors may ultimately enhance maternal and neonatal outcomes, contributing to improved quality of life for mothers, infants, families, and society at large. We also wish to highlight that, to our knowledge, multiclass classification of birthweight outcomes remains relatively underexplored in the literature, despite its greater clinical relevance compared to binary classification. Specifically identifying VLBW cases enables more targeted treatment planning and efficient resource allocation, which can help prevent severe neonatal complications such as sepsis or respiratory distress. Most existing research in this domain relies on binary classification, typically combining LBW and VLBW infants into a single category (< 2500 g). However, given the substantial differences in clinical management between LBW and VLBW infants, we aimed to develop a model capable of distinguishing VLBW cases (< 1500 g). Although our model’s current predictive accuracy for VLBW remains limited, we view this work as a meaningful step toward addressing a critical research gap and supporting future advancements in this area.

Our study has several limitations. First, the information was gathered exclusively from individuals of Indian ethnicity, making the findings applicable only to pregnant Indian women and limiting the generalizability of the model. To enhance the classifier’s reliability, datasets from various ethnicities should be included in future research. This study employed supervised learning methods, excluding unsupervised and reinforcement learning algorithms. We acknowledge that our sample size was limited, consisting of only 237 pregnant women with a data imbalance, particularly in VLBW infants. Although we initially aimed for a sample of 356, only 237 women delivered at our institution, primarily due to the socio-cultural practice of giving birth at their maternal home. While transfer learning could potentially address issues related to sample size, we opted not to rely on it extensively in this study due to computational limitations and our focus on XAI for better interpretability. Furthermore, external validation of the model was not conducted for ethical reasons, as well as due to the nature of the data used in this study. This lack of validation limits the generalizability of the model across diverse populations and clinical settings. However, we did perform cross-validation by separating the dataset into training and test sets. Therefore, a larger sample size that includes diverse medical conditions and ethnicities would strengthen the robustness of the model.

## Conclusion

The current study used multiple supervised learning algorithms and XAI techniques to predict birthweight using early and mid-pregnancy clinical markers. The developed model may help medical practitioners make more accurate birthweight predictions that are readily available using routine antenatal features. Furthermore, the integration of a cloud database incorporating sociodemographic, genetic, clinical, antenatal, and postnatal data can support advancements in fetal precision medicine.

## Data Availability

The datasets generated and analysed during the current study are not publicly available due to the sensitive nature of the data but are available from the corresponding author on reasonable request.
